# Understanding the unfolding of stress regulation in infants

**DOI:** 10.1017/S0954579416000171

**Published:** 2016-03-29

**Authors:** HEIDEMARIE K. LAURENT, GORDON T. HAROLD, LESLIE LEVE, KATHERINE H. SHELTON, STEPHANIE H. M. VAN GOOZEN

**Affiliations:** a University of Oregon; b University of Sussex; c Tomsk State University; d Cardiff University; e Leiden University

## Abstract

Early identification of problems with psychosocial stress regulation is important for supporting mental and physical health. However, we currently lack knowledge about when reliable individual differences in stress-responsive physiology emerge and which aspects of maternal behavior determine the unfolding of infants’ stress responses. Knowledge of these processes is further limited by analytic approaches that do not account for multiple levels of within-and between-family effects. In a low-risk sample (*n* = 100 dyads), we observed infant cortisol and mother/infant behavior during regular play and stress sessions longitudinally from age 1 to 3, and used a three-level model to separately examine variability in infant cortisol trajectories within sessions, across years, and across infants. Stable individual differences in hypothalamus–pituitary–adrenal axis regulation were observed in the first 3 years of life. Infants of less sensitive and more intrusive mothers manifested stress sensitization, that is, elevated cortisol levels during and following stress exposure, a profile related to behavioral distress. These findings have important practical implications, suggesting that children at risk for long-term stress dysregulation may be identified in the earliest years of life.

Individual variability in the likelihood that specific stressors are linked to stress responses plays a key role in mental and physical health (e.g., Cacioppo, 1998; Rogosch, Dackis, & Cicchetti, 2011; Dunkel Schetter & Dolbier, 2011), making early identification of problematic responding a priority. However, such efforts are hampered by a lack of knowledge about when reliable individual differences in stress-responsive physiology emerge. It is also well known that the quality of maternal care predicts child neuroendocrine self-regulation outcomes (e.g., Adam, Klimes-Dougan, & Gunnar, 2007; Bugental, Martorell, & Barraza, 2003; Cicchetti & Rogosch, 2001), but less is known about precisely how this occurs, that is, which aspects of maternal behavior influence the unfolding of infants’ stress responses, and how these physiological responses map onto behavioral adjustment. In this report, we use a multilevel approach to a longitudinal study of mother–infant dyads to shed light on the stability and variability in functioning of the hypothalamus–pituitary–adrenal (HPA) axis across the first years of life, as well as linkages with observed maternal and infant behaviors. With this work, we address unanswered questions about when adjustment-relevant individual differences in stress responding can be detected, and specify dimensions and contexts of maternal behavior most likely to influence stress regulation development.

## The HPA Axis as a Marker of Stress Regulation

As one of the major branches of the stress response system, the HPA axis, whose activity is typically measured through salivary cortisol in humans, prepares the organism to respond to sustained psychological and/or physical threat by modulating metabolic, immune, and cognitive functions (McEwen, 2007; Sapolsky, Romero, & Munck, 2000). A well-regulated HPA response plays a vital role in psychobiological adaptation to stress, and there is ample evidence that variations in HPA reactivity and recovery act as a mechanism linking adversity exposure with poor mental and physical health outcomes (e.g., Gunnar & Vazquez, 2001; Koss et al., 2013; Lopez-Duran, Kovacs, & George, 2009; Sturge-Apple, Davies, Martin, Cicchetti, & Hentges, 2012). However, there is ongoing disagreement about the type of HPA response that signals risk; whereas many of these studies point to elevated and/or extended cortisol responses (stress “sensitization”) among children raised in adverse environments who go on to show behavioral problems, others point to blunted responses to psychosocial stress. The differences may have to do with the intensity and/or timing of exposure, with researchers proposing that particularly intense chronic stress early in development can give rise to initial HPA hyperactivation followed by downregulation (see Doom, Cicchetti, & Rogosch, 2014). At this point, it is important to note that *regulation* can be defined in different ways. Here, we acknowledge that a variety of stress response profiles may emerge as attempts to optimize survival in nonoptimal caregiving environments, and in this sense they have an adaptive value. At the same time, these adaptations may carry behavioral costs, that is, heightened distress when confronted with stress, longer term internalizing and externalizing symptoms, which negatively impact developmental trajectories. Thus, we operationalize HPA regulation in this paper as cortisol profiles most likely to support behavioral adjustment, based on associations with (lower) distress.

The above research spans a range of developmental periods, yet there is reason to believe that the earliest years offer a critical window into stress regulation processes. Not only are neurophysiological systems undergoing rapid development, but also the impacts of the caregiving environment are known to be particularly salient during this time (e.g., Cicchetti & Curtis, 2006; Levine, 2005; Tarullo & Gunnar, 2006). To better understand the precursors and nature of HPA regulation as it emerges, it is important to consider care-giver effects on cortisol responsiveness during infancy.

## The Role of Maternal Behavior

In the earliest phases of human development, the mother is thought to act as an external regulator of infant arousal; the mother is attuned to and acts to soothe distress during a period when the infant has not yet developed an extensive repertoire of self-regulatory capacities (see Hofer, 1995). This sensitive caregiving style, characterized by an accurate interpretation of and prompt response to infant needs, protects infants from excessive stress and allows them to develop effective stress regulation (e.g., hippocampal control of HPA axis activity). However, mothers vary in the degree to which they fulfill this function, with some showing less positive (i.e., sensitive engagement) and/or more negative (i.e., intrusiveness) behaviors with their infants that lead to poorer social–emotional development (e.g., Feldman, 2010). Although there is general agreement about which maternal behaviors are beneficial versus harmful, more work needs to be done to determine precisely which dimension/s of behavior (sensitivity or intrusiveness) and which interaction context/s (unstructured play or stressful events) are most critical for developing HPA regulation.

Both sensitive and intrusive maternal behaviors during stressful and free-play interactions have been associated with child physiological and/or behavioral self-regulation outcomes (see DiCorcia & Tronick, 2011; Hostinar & Gunnar, 2013). Behavioral research tends to emphasize the importance of maternal sensitivity in the context of stress/distress for a variety of child adjustment outcomes (e.g., Leerkes, Blankson, & O’Brien, 2009; Manning, Davies, & Cicchetti, 2014; McElwain & Booth-LaForce, 2006). There is also some research suggesting a causal role of maternal sensitivity in infant HPA axis regulation (Cicchetti, Rogosch, Toth, & Sturge-Apple, 2011), but there is still not enough comparative research involving a range of maternal behaviors and infant HPA function during acute stress to determine whether this holds for the physiological domain.

Another limitation to the existing literature is that studies showing effects of maternal behaviors on infant HPA activity tend not to include measures of observed child behaviors. Given conflicting findings for the adjustment value of high versus low cortisol levels referred to above, it is important to determine how infant HPA profiles related to maternal behaviors compare to those related to infant behavioral adjustment to be able to characterize a given maternal influence as regulating. Finally, very little is known about the temporal nature of maternal and/or infant behavior effects, that is, whether these represent stable individual differences or changing proximal influences on infant stress physiology. The demands on maternal sensitivity are expected to change across development, as well as across child emotion contexts, and shifts in maternal behaviors undetected by a single behavioral assessment may play a key role in infant regulatory capacities (see Thompson, 1999). In order to identify and intervene to help infants at risk, it is important to fully understand the developmental underpinnings of stress regulation.

## Early Development of Stress Regulation

A review of (mostly cross-sectional) infant cortisol research suggests inconsistent reactivity to psychosocial stress, with a general decline across the first several years of life (Jansen, Beijers, Riksen-Walraven, & de Weerth, 2010). Limited longitudinal research similarly points to instability in infants’ cortisol responses and inconsistent relations with maternal factors over time (Tollenaar, Beijers, Jansen, Riksen-Walraven, & de Weerth, 2011, 2012). One study that addressed both maternal and infant behaviors in relation to cortisol showed that maternal engagement related to greater infant cortisol reactivity at 7 months, but lower overall cortisol levels at 15 months (Blair et al., 2008). At 15 months child distress to novelty was associated with increased cortisol reactivity and regulation, whereas distress to limitations was associated with reduced cortisol reactivity. Martinez-Torteya et al. (2015) recently found that infants did not show a cortisol response at 7 months, but reactivity to psychosocial stress emerged by 16 months. Individual differences in cortisol baseline and reactivity levels over time were found to be related to infant sex and maternal overcontrolling behaviors, underscoring, according to the authors, the malleable and socially informed nature of early HPA axis functioning. These inconsistencies may speak to developmental characteristics of the HPA axis, but they may also have to do with different types of stressors employed at different ages and relatively simplistic data analytic approaches (i.e., correlations) that do not distinguish between-family differences from within-family influences over time.

A longitudinal study of children’s diurnal cortisol levels from age 9 to 15 years employed multilevel modeling to determine the presence of traitlike stability in HPA activation, showing that differences in trait cortisol and covariation with child symptoms were related to the early parenting environment (Essex et al., 2011). A similarly nuanced approach to HPA function in infancy is needed to determine whether trait-like variation can be detected earlier in development, and how this intersects with maternal and child behaviors. To our knowledge, no previous research has examined long-term (across multiple years) development of infant HPA responses, nor distinguished between- versus within-family level effects on these responses.

## The Current Study

The current investigation, conducted in a sample of infants and their mothers assessed longitudinally during stress sessions from age 1 to 3, had two primary goals. First, we aimed to identify sources of infant HPA variability early in development, in particular, to establish whether reliable individual differences in HPA responses can be discerned during the first several years of life, and how normative HPA responses evolve during this period. Second, we aimed to clarify the nature of associations between infant HPA function and both maternal and infant behavior over time. Specifically, we employed a multilevel approach not yet applied to early HPA axis development to clarify (a) the importance of maternal sensitivity versus intrusiveness for infant cortisol levels during stress versus free-play interactions, (b) whether these effects parallel associations with infant distress, and (c) whether each of these associations can be best explained by stable between-family differences or variability in behavior over time.

## Method

### Participants

Participants were 100 mother–infant dyads recruited from local nurseries and leisure centers. Infants (49 males, 51 females) were recruited around their first birthday (mean age =10.01 months, *SD* = 1.76, range =9–13), and returned to the laboratory within 1 month of their second and third birthdays. Sample size was determined by practicalities relating to participant recruitment and assessment. A minimum sample of >80 participants (across all waves and methods of assessment) was estimated a priori to provide adequate statistical power for all hypothesized primary statistical analyses.

Mothers were aged 22 to 43 years old (mean = 33.43 years, *SD* = 4.47) at the first assessment. Ninety-one percent of mothers were married and 9% were single. The majority of mothers had university degree level education (78.2%) with over half of these holding postgraduate qualifications (i.e., master’s or higher, 41%). Smaller numbers of mothers held secondary school level qualifications only (i.e., 6.4%), or had completed high school (3.8%) or diploma level training (11.5%) only. Families were predominantly of White British origin (87.2%), with smaller proportions of participants of Asian Indian (9%), Black (2.6 %), and Middle Eastern origin (1.3%). All the infants had been full-term and just over half of the infants were firstborn (54%). Ethical approval for the study was obtained from Cardiff University’s School of Psychology Research Ethics Committee.

### Procedures and measures

#### Maternal behavior

Maternal sensitivity and intrusiveness were assessed as separate constructs during two interactions, occurring at all three assessment waves. The first interaction was when the mother and infant were alone in a child-friendly playroom and asked to play together with a standard selection of toys. We refer to this interaction as the *free-play* interaction. The second interaction was part of and followed an infant fear challenge task, which consisted of the unpredictable mechanical toy episode of the Laboratory Temperament Assessment Battery (Goldsmith & Rothbart, 1999). The mother was asked to leave the room during the fear challenge (Baker, Baibazarova, Ktistaki, Shelton, & van Goozen, 2012; Baker, Shelton, Baibazarova, Hay, & van Goozen, 2013), and mother–child interaction was assessed upon reunion. We refer to this as the *stress* interaction.

Each mother–infant interaction lasted 3 min and was recorded on videotape. All videotapes were scored after the three waves of the study had been completed. Maternal behavior was assessed on parameters of maternal sensitivity and maternal intrusiveness using the scoring system developed by Fish and Stifter (1995). Both behaviors were rated at 30-s intervals on 4-point (0–3) scales designed to reflect none, a low level, a moderate level, or a high level during each 30-s period (i.e., six scores for each interaction). The summed scores for maternal sensitivity or maternal intrusiveness during one interaction episode could range from 0 to 18. The interrater reliability (Cohen κ) between two trained coders on 11% of the sample ranged from 0.71 to 0.73 for maternal sensitivity and from 0.73 to 0.78 for maternal intrusiveness across waves. These values concur with reliability scores for other studies of observed maternal behavior in laboratory conditions (e.g., Kok et al., 2013).

#### Infant distress

Infant temperamental distress was assessed following the Laboratory Temperament Assessment Battery’s guidelines for the behavioral coding of episodes, using video recordings of the session (Goldsmith & Rothbart, 1999). The episode lasted approximately 3.5 min. Each of the three trials of unpredictable toy approach was separated into three epochs, creating a total of nine epochs that were scored separately. Each epoch was scored on the following dimensions and scales: intensity of facial fear (0–3), intensity of facial sadness (0–3), intensity of distress vocalization (0–5), intensity of bodily fear (0–3), intensity of escape (0–3), and presence/absence of startle response (0–1). The high reliability between these variables (Cronbach *α* = 0.84) enabled us to create a composite score by adding the individual ratings for these temperament variables across the distress episode to indicate an overall level of temperamental distress (Baker et al., 2013). The possible range for the composite score was 0 to 162. Four coders scored the episodes independently. Intracorrelation coefficients between coders ranged between 0.70 and 0.99 across the behavioral variables for 11% of the sample.

#### HPA axis activation

In order to measure physiological stress, salivary samples for the assessment of cortisol were collected from each infant at each wave, including two baseline and two (Wave 1) or three (Waves 2 and 3) poststress samples. The first baseline saliva sample was taken shortly after mother and infant’s arrival at the laboratory (Sample 1 taken at 9.15 a.m.); the second baseline sample was collected 15 min later (Sample 2 at 9.30 a.m.). The first poststress sample was taken 20 min after the start of the distress challenge (Sample 3 at 10.20 a.m.), and the fourth and fifth samples were taken 25 (at 10.45 a.m.) and 45 min (at 11.05 a.m.) after the third. Each sample collection took approximately 1 min. Sorbettes and cryovials (Salimetrics, State College, PA) were used for collecting saliva from the infant’s mouth. Because of the evidence that milk can interfere with the cortisol assay (Maganon, Diamond, & Gardner, 1989) mothers were asked not to feed their infants during the assessment. After collection, samples were frozen at −20 °C and stored until they were shipped in dry ice for analysis. All samples were analyzed with ELISA cortisol assays. The samples were spun at 15,000 rpm for 15 min at 4 °C and assayed in duplicate. The data were transferred to a computer using the assay software KC4, creating a standard curve. The concentration of cortisol present in each sample was then calculated from the standard curve. A standard curve was generated for every plate of samples assayed. The average intra- and interassay coefficients of variation were 4.33% and 9.25%, respectively. Table 1 shows means and standard deviations for cortisol samples across years. There were no sex differences in cortisol trajectories, and thus we did not include sex in subsequent analyses.

### Analytic approach

Multiple imputation in MPlus was used to estimate missing maternal and infant behavior scores; mean scores from five generated data sets were used in analyses. Missing data analysis was conducted by comparing dyads with missing cortisol data at Time on each of the infant fear and mother sensitivity/intrusiveness variables. There was no evidence of significant differences based on patterns of missingness at Time 1. Hierarchical linear modeling (HLM) was selected to capture associations within a nested data structure, that is, cortisol scores nested within sessions within infants. In particular, a three-level model was used to separately examine (a) variability in infant cortisol within sessions (Level 1), (b) variability in infant cortisol across years (Level 2), and (c) variability in cortisol trajectories across infants (Level 3). At the first level, each infant’s cortisol scores within each session were fit to a quadratic model to reflect the expected pattern of reactivity and recovery across the session. Models were centered at the peak stress sample so that intercepts reflected the infant’s level of peak stress cortisol, slopes reflected the infant’s instantaneous rate of reactivity or recovery at that point of peak stress, and quadratic terms reflected the steepness of the infant’s overall reactivity/recovery curve.

At the second level, variation in these terms across years was modeled with an intercept and linear slope. Level 2 models were centered at the first assessment so that intercepts reflected the infant’s cortisol (intercept/slope/quadratic) at Year 1, and slopes reflected change in that parameter across the 3 years. Finally, variation in these Level 2 terms was modeled at the third level.

Maternal behavior (sensitivity or intrusiveness during free-play and stress periods) was used to predict infant cortisol at multiple levels. At Level 2, maternal behavior was entered as a group mean-centered predictor of Level 1 trajectory terms; that is, the mother’s mean behavior score for that session was used to predict the infant’s cortisol intercept/slope/quadratic at that session. The centering meant that positive values represented years when the mother’s behavior was higher than her own average, and negative values represented years when her behavior was lower than her own average, across the three sessions. Mean maternal behavior across all assessments was then entered as a grand mean-centered Level 3 predictor of infant cortisol intercepts and slopes at Level 2. This tested whether infants of mothers who were more sensitive or intrusive overall showed differences in Year 1 stress physiology trajectories and change across years.

In addition to the main modelstesting associations with maternal behavior, another set of models examined associations with observed infant distress behavior during sessions. These models helped to contextualize the primary model results by clarifying which cortisol patterns marked more fearful infants.

## Results

Means, standard deviations, range, and *n*’s for all study variables are shown in Table 1; correlations among maternal and child variables are shown in Table 2. Correlations among mother and child behaviors reflected expected patterns; maternal sensitivity and intrusiveness were positively related across free-play and stress periods, and inversely related to one another. Child distress was related to lower maternal sensitivity during stress only (see Table 2).

### Preliminary models

Baseline HLM models containing no behavior predictors were fit to determine the best way to model infant cortisol trajectories. Cortisol scores were log-transformed prior to analysis to correct positive skew. The best fitting model, according to change in the deviance statistic, incorporated quadratic trajectories within sessions, with slopes of linear change in these trajectories across years, χ^2^ (12) = 116.26, *p* < .001, for adding quadratic trajectory term at Level 1; χ^2^ (18) = 51.1, *p* < .001, for adding linear slope term at Level 2. According to the baseline model, cortisol levels (intercepts) decreased normatively across years (β = −0.34, *p* < .001). Cortisol showed significant variability (according to the tau statistic) at all three levels of modeling: 25% at Level 1, 34% at Level 2, and 41% at Level 3.

To better understand how maternal behavior predictors varied over time, sensitivity and intrusiveness were also examined using three-level HLM models. These models offered evidence for developmental stability, with approximately one third of the variance at the between-mother level (Level 3; 32% for sensitivity, 37% for intrusiveness), and a smaller proportion attributable to variation across years (Level 2; 16% for sensitivity, 10% for intrusiveness). At the same time, significant coefficients for yearly change suggested that mothers normatively became more sensitive and less intrusive across years (β = 0.20, *p* = .001 for sensitivity; β = −0.29, *p* < .001 for intrusiveness). Over half of the variance in maternal behavior derived from within-session changes (52% for sensitivity, 53% for intrusiveness). Repeated measures *t* tests showed that mothers were, on average, more sensitive and less intrusive during stress compared to free-play periods, *t* (99) = 2.73, *p* = .007 for sensitivity; *t* (99) = 6.32, *p* < .001 for intrusiveness. Whereas the difference in intrusiveness was evident across years, the difference in sensitivity only became significant at the final assessment year.

### Explanatory models: Maternal sensitivity

As described above, maternal sensitivity during free-play and stress periods was entered at Levels 2 and 3 to predict infant cortisol. The overall maternal sensitivity during both free-play and stress related to lower cortisol levels and quicker recovery (more negative slope and quadratic terms; Table 3). These effects on cortisol dynamics, that is, slope and/or quadratic terms, but not intercepts, tended to become more moderate across years.

### Explanatory models: Maternal intrusiveness

**Figure 1:**
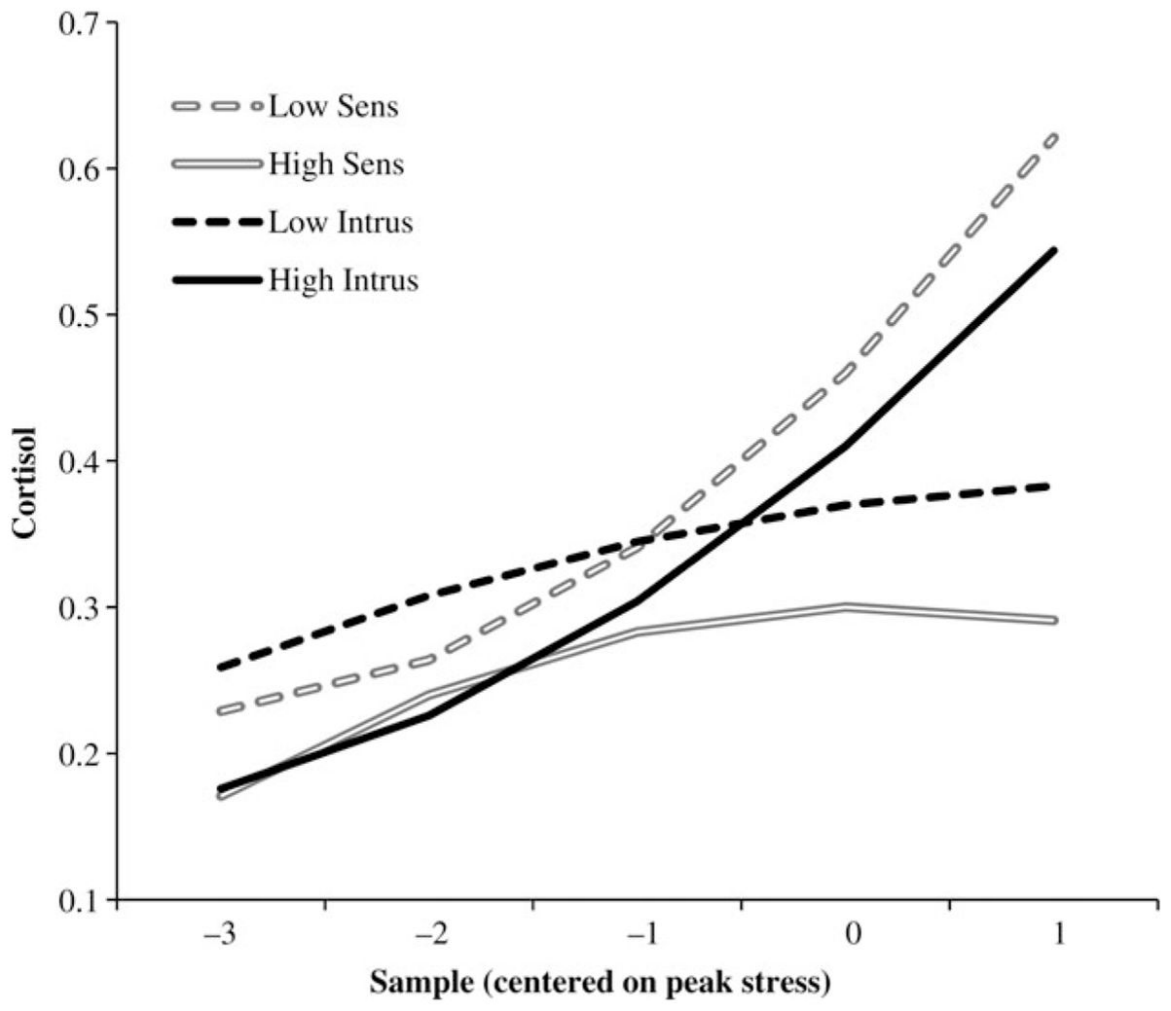
Child cortisol trajectories related to maternal behaviors (Level 3 effects). The values are the predicted trajectories at high (+1 *SD*) and low (−1 *SD*) values of mean maternal behavior during free-play interaction. Sens, sensitivity; Intrus, intrusiveness.

Maternal intrusiveness during free-play and stress periods was entered to predict infant cortisol, typically yielding opposite effects to those found for sensitivity. At Level 2, higher intrusiveness during stress related to a flatter cortisol curve (more positive quadratic) across years (Table 3). At Level 3, higher intrusiveness during free-play related to slower cortisol recovery (more positive slope; Table 3). Again, this effect tended to become more moderate across years. Figure 1 depicts between-child maternal sensitivity and intrusiveness effects.

### Explanatory models: Infant temperamental distress

**Figure 2:**
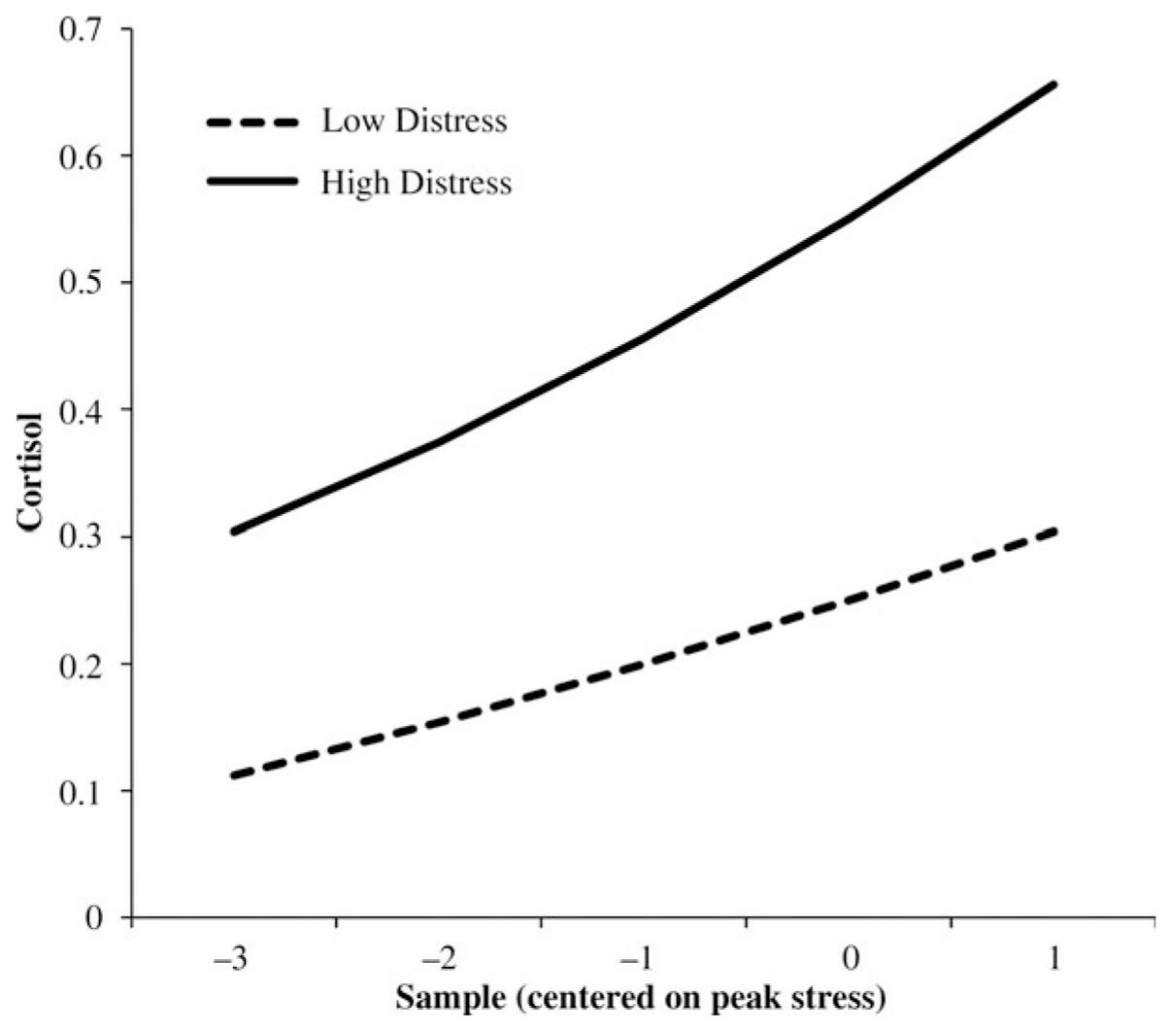
Child cortisol trajectories related to fearful behavior (Level 3 effects). The values are the predicted trajectories at high (+1 *SD*) and low (−1 *SD*) values of mean child behavior.

Infant temperamental distress related to higher cortisol levels at Year 1 and an increase across years, as well as slower cortisol recovery (positive slope) across years (Table 4). Figure 2 shows between-child differences in cortisol trajectories related to distress.

### Follow-up tests: Unique versus shared effects

Given that maternal behaviors related to one another, and at least one of these (sensitivity during stress) related to infant behavior, models including multiple behavior predictors were run to ascertain the degree to which shared versus unique variance contributed to the above effects. When concurrent maternal sensitivity and intrusiveness were included together, several of the free-play behavior effects (sensitivity predicting linear and quadratic terms; intrusiveness predicting linear term) were no longer significant, though the coefficients did not change markedly (within 98% confidence interval of original estimates). All stress behavior effects, in contrast, remained significant. Similarly, when free-play and stress measures of sensitivity or intrusiveness were included together, the stress effects proved most important (free-play behavior effects became nonsignificant and were reduced in size). Finally, maternal and infant behavior effects remained unchanged when included together.

### Summary

In summary, the above models demonstrated that there is meaningful variability in infants’ stress physiology within sessions, across years, and between infants. More sensitive mothers had infants who displayed better stress regulation, that is, lower cortisol with quicker poststress recovery, whereas intrusive mothers had the opposite effect. These effects were most evident for maternal behavior during stress and at the between-infant (family) level of analysis early in development. Finally, we found at least some evidence that HPA hyperactivation, that is, consistently high, nonrecovering cortisol, related to infant distress.

## Discussion

In the first investigation of its kind that we know of, we show that stable individual differences in HPA regulation can be observed in the first 3 years of life. We further clarify the importance of maternal influences by showing that infants of less sensitive, more intrusive mothers evidence dysregulation, that is, elevated cortisol levels during and following stress exposure, a profile related to behavioral distress. These findings have important practical implications, suggesting that children at risk for long-term problems with stress regulation may be identified in the earliest years of life. Notable elements of the current study’s design, including multilevel analysis of infant cortisol before and after the same stress task across multiple years, may have allowed us to detect stability not found in previous infant research.

This study supports the contention, based largely on behavioral research, that maternal sensitivity during stress is especially crucial for the development of stress regulation. Although maternal sensitivity during free-play periods, as well as intrusiveness, also played a role in infants’ HPA function, these effects were less widespread. As argued by attachment researchers, the caregiver’s ability to respond promptly and appropriately to distress cues constitutes a crucial organizer of the infant’s developing capacity to downregulate negative arousal and safely engage with novel stimuli. Most mothers appeared to shift their behavior to facilitate this process, increasing sensitivity and reducing intrusiveness during stress (relative to free-play) periods. Shifts were also apparent over the longer time scale of infant development, with mothers normatively becoming more sensitive and less intrusive from the first to third postnatal year. This specificity suggests researchers should distinguish maternal behavior effects based on the interaction context, rather than assuming that a single (free-play) measure offers the information needed to understand infant outcomes. Future research should explore how the relative importance of sensitivity versus intrusiveness in different types of interactions may change with development, guiding recommendations for parenting interventions.

The correspondence between infant cortisol profiles associated with maternal insensitivity and infant distress helps make the case that sensitive mothers can be considered “regulating.” There was no evidence in this sample for a mediated effect (i.e., maternal sensitivity impacting infant cortisol via distress); rather, maternal and infant behaviors each related uniquely to infant cortisol. Further work will be needed to determine underlying processes, which may involve moderated paths (i.e., differential effects of maternal sensitivity based on child temperament) as proposed by differential susceptibility theory (Belsky & Hartman, 2014; see also Sturge-Apple et al., 2012). For now, that HPA hyperactivation characterized both temperamentally distressed infants and those with insensitive mothers strengthens the argument that this represents a dysregulated phenotype. These findings also lend support to a stress sensitization (rather than downregulation) model of the effects of mild adversity exposure during infancy.

Our sample was representative of mothers and young children living in a community, UK setting, and as such did not represent a specific at-risk grouping. This offers a distinct strength in examining a range of stress-related responses in infants relative to maternal behaviors. In order to better understand stress responses and related mechanisms in the context of risk (abnormal developmental processes), it is first necessary to examine and quantify such processes in the context of normal development. The current results in this low-risk sample help us to better understand how normative developmental processes in typically developing children may go awry when they occur in the context of elevated maternal insensitivity or infant temperamental distress and exacerbate stress-sensitization processes. These profiles in normal healthy samples can extend into psychopathological patterns, and the early detection of more extreme variations in reactivity in very young children may ultimately have implications for the prevention of both internalizing and externalizing disorders. Replication of these findings in high-risk families and extension of the identified processes to predict indicators of psychopathology are the logical next steps.

Multilevel modeling, which separated person-level, age-related, and within-session effects, suggested maternal behavior effects were largely attributable to stable individual differences, rather than time-specific variations. At the same time, we observed more marked effects of maternal behavior early on that became more moderate across the 3 years of study. It may be that normative changes in both maternal behavior (i.e., more sensitive/less intrusive) and infant physiology (less reactive) lead to muted impacts across the infancy period. Given within-child stability of cortisol profiles, this argues for the importance of early intervention to help mothers develop skills that will provide acrucial foundation for child regulation. In particular, prenatal intervention with women at risk for parenting problems could have far-reaching impacts (Smaling et al., 2015).

In addition to the strengths of this study, that is, the multi-year longitudinal design with the same protocol at three occasions, multiple assessments of both maternal behavior and infant cortisol in relation to stress at each occasion, limitations should be considered in the interpretation of results and used to suggest avenues for future research. Like many other studies of maternal sensitivity and child development, our sample was generally low risk, White, and well educated. There is important work still to be done to examine the extent to which these findings generalize to high-risk families, particularly those experiencing maternal psychopathology, family conflict, and socioeconomic pressure. While some dynamic shifts in mother–infant interactions might be optimal for fostering emotion regulation where the mother is established as a source of security and safety, greater consistency in response may be optimal in environments more subject to change, or characterized by lower levels of positive stimulation and even danger. Another next step is to examine factors that may affect maternal behavior such as father involvement and pressures at work. Expanding models in terms of both scope (beyond observed maternal behaviors) and time (beyond infancy) promise to further illuminate paths to child (dys)regulation and inform early intervention efforts.

This study sheds new light on the early roots of stress regulation as a dynamic, socially guided process that impacts basic biological functions. It is our hope that this information will help refine efforts to identify and treat families at risk for stress-related difficulties before these become entrenched.

## Figures and Tables

**Table 1:** Descriptive information for cortisol and behavioral variables across years

Variable	Age 1				Age 2				Age 3			
	*M*	*SD*	Range	*n*	*M*	*SD*	Range	*n*	*M*	*SD*	Range	*n*
Cortisol 1	3.26	6.72	0.14–40.35	98	1.39	1.49	0.10–11.66	63	1.59	3.80	0.10–29.02	62
Cortisol 2	3.10	6.39	0.29–49.67	96	2.63	7.08	0.29–45.01	60	1.78	4.45	0.10–34.48	60
Cortisol 3	3.07	4.48	0.45–34.16	96	2.59	4.43	0.10–27.68	67	1.59	1.48	0.10–7.78	62
Cortisol 4	4.65	7.69	0.14–50.00	94	2.86	2.78	0.15–12.77	64	2.06	1.33	0.14–5.87	61
Cortisol 5	—		—	—	1.80	1.20	0.10–6.33	39	1.73	1.71	0.10–10.17	59
Child distress	60.35	37.48	5–142	100	82.00	42.42	8–153	80	77.94	46.98	0–162	71
Mater. sensitiv.												
Free play	10.44	2.48	6–17	68	11.48	1.88	5–15	61	10.98	2.32	6–17	52
Stress	10.63	2.62	5–17	73	11.98	2.22	3–17	60	11.91	1.95	7–17	54
Mater. intrusive.												
Free play	3.82	3.37	0–13	68	1.84	2.77	0–15	61	2.37	2.56	0–11	54
Stress	1.54	1.88	0–7	72	1.16	1.94	0–8	59	0.52	1.49	0–10	54

*Note:* Raw scores are shown. Variables were log transformed as necessary to correct for skew prior to analysis.

**Table 2:** Correlations among maternal and child behaviors

	1	2	3	4	5
1. Mater. sensitiv., free play	—	**.40**	**−.35**	**−.22**	−.02
2. Mater. sensitiv., stress	**.45**	—	**−.25**	**−.26**	**−.17**
3. Mater. intrusiv., free play	**−.50**	**−.26**	—	**.34**	−.10
4. Mater. intrusiv., stress	**−.42**	**−.33**	**.37**	—	.004
5. Child distress	–.07	**−.39**	–.06	.07	—

*Note:* Level 2 (yearly) effects are above the diagonal and Level 3 (overall mean) effects are below the diagonal. Significant correlations (*p* < .05) are highlighted in bold.

**Table 3:** Associations between maternal behavior and child cortisol

	Intercept		Slope		Quadratic	
	β	95% CI	β	95% CI	β	95% CI
Level 3 (between child) effects						
1. Sensitivity, free play						
Effect on Year 1 level	**−0.21**	**−0.39 to −0.03 (14.7%)**	**−0.18**	**−0.31 to −0.05 (13.3%)**	**−0.04**	**−0.08 to −0.005 (29.6%)**
Effect on Year 1–3 slope	0.03	−0.10 to 0.16	**0.12**	**0.05 to 0.19 (22.2%)**	**0.04**	**0.01 to 0.06 (38.1%)**
2. Sensitivity, stress						
Effect on Year 1 level	**−0.31**	**−0.49 to −0.13 (23.5%)**	**−0.21**	**−0.33 to −0.09 (24.4%)**	**−0.05**	**−0.08 to −0.02 (37.0%)**
Effect on Year 1–3 slope	0.07	−0.06 to 0.20	**0.12**	**0.05 to 0.19 (38.9%)**	**0.04**	**0.01 to 0.06 (38.1%)**
3. Intrusiveness, free play						
Effect on Year 1 level	0.05	−0.13 to 0.23	**013**	**0.01 to 0.25 (16.7%)**	0.02	−0.02 to 0.06
Effect on Year 1–3 slope	−0.02	−0.14 to 0.10	−0.07	−0.15 to 0.008	−0.02	−0.05 to 0.009
Level 2 (within-child) effects						
4. Intrusiveness, stress						
Yearly effect	−0.11	−0.34 to 0.12	0.15	−0.007 to 0.31	**0.06**	**0.001 to 0.12 (0%)**

*Note:* Significant effects (*p* < .05) are highlighted in bold. The model term’s percentage variance is in parentheses.

**Table 4:** Associations between child behavioral distress and cortisol

	Intercept		Slope		Quadratic	
	β	95% CI	β	95% CI	β	95% CI
Level 3 (between child) effects						
Effect on Year 1 level	**0.39**	**0.22 to 0.56**	0.06	−0.05 to 0.17	0.004	−0.03 to 0.04
	**(26.5%)**					
Effect on Year 1–3 slope	−0.10	−0.22 to 0.02	−0.04	−0.11 to 0.03	−0.01	−0.04 to 0.02
Level 2 (within-child) effects						
Yearly effect	**0.21**	**0.08 to 0.34**	**0.10**	**0.01 to 0.19**	0.01	−0.02 to 0.04
	**(9.8%)**		**(6.9%)**			

*Note:* Significant effects (*p* < .05) are highlighted in bold. The model term’s percentage variance is in parentheses.
